# Pecan nut–derived carbon quantum dots–modified electrode for sensitive electrochemical determination of vonoprazan using a green analytical approach

**DOI:** 10.1038/s41598-026-68500-y

**Published:** 2026-09-08

**Authors:** Arwa Gamal Elbanna, Aya Barseem, Hytham M. Ahmed

**Affiliations:** 1 Pharmaceutical Analysis Department, Faculty of Pharmacy, Menoufia University, Shibin El Kom, Egypt; 2 Department of Pharmaceutical Analytical Chemistry, Faculty of Pharmacy, Menoufia National University, 70 Km Cairo-Alexandria Agricultural Road, Menoufia, Egypt

**Keywords:** Voltammetry, Electrochemical sensor, Pecan nut (*Carya illinoinensis*)–derived CQDs, Vonoprazan, Green analytical chemistry, Chemistry, Environmental sciences, Materials science, Nanoscience and technology

## Abstract

A novel, environmentally considerate electroanalytical sensor was developed for the determination of vonoprazan fumarate (VON) through modification of a carbon paste electrode with biomass-derived carbon quantum dots (CQDs), representing a sustainable, defect-rich, and effective alternative to conventional CQD-based sensors that rely on synthetic materials and complex fabrication. The CQDs were synthesized from pecan nut (*Carya illinoinensis*) using a microwave-assisted solvothermal approach and exhibited abundant functional groups and quantum confinement effects, as confirmed via X-ray photoelectron spectroscopy (XPS) in combination with Fourier-transform infrared (FTIR) spectroscopy, fluorescence spectroscopy, ultraviolet–visible spectroscopic approach (UV–Vis), transmission electron microscopy (TEM) and scanning electron microscopy combined with energy-dispersive X-ray spectroscopy (SEM–EDX). Electrochemical impedance spectroscopy (EIS) revealed enhanced interfacial electron-transfer kinetics and electrocatalytic activity, highlighting the enhanced electrode performance of the CQD-modified sensor and its direct contribution to improved sensitivity and selectivity. Quantitative determination of VON was performed using differential pulse voltammetry (DPV), achieving a limit of detection of 0.39 nM, a limit of quantification of 1.19 nM, and a linear response over 10–100 nM (R^2^ = 0.9992). The method met international validation criteria for accuracy, precision, and robustness, and was successfully applied to pharmaceutical preparations, spiked human plasma samples, and laboratory-prepared industrial wastewater. This work addresses the growing demand for sensitive, reliable, and eco-friendly detection of pharmaceutical analytes, providing a versatile, sustainable, and effective sensing platform.

**Supplementary Information:**

The online version contains supplementary material available at https://doi.org/10.1038/s41598-026-68500-y.

## Introduction

Gastric acid-mediated gastrointestinal pathologies, including Helicobacter pylori infection, peptic ulcer disease (PUD), and gastroesophageal reflux disease (GERD), pose a significant healthcare burden due to their high prevalence and potential for severe complications. GERD arises from reflux of gastric contents, leading to heartburn, regurgitation, and chronic mucosal injury, while PUD involves erosive lesions in the gastric or duodenal lining, potentially causing pain, bleeding, or perforation. Persistent H. pylori infection exacerbates disease progression and increases the risk of gastric malignancy. Effective therapy and clinical monitoring of these disorders require reliable analytical tools for precise drug quantification in diverse biological and pharmaceutical matrices^1–5^.

Vonoprazan fumarate (VON) is a recently developed potassium-competitive agent that reversibly inhibits the gastric H^+^/K^+^-ATPase proton pump, offering rapid onset, acid stability, and prolonged pharmacological activity. Its growing clinical adoption underscores the need for sensitive and selective analytical platforms capable of trace-level detection^6–10^. Conventional chromatographic techniques, such as LC–MS^11–13^, HPLC^14–16^, RP-HPLC^17–19^, and UHPLC^20^, provide reliable analytical performance but require complex instrumentation and extensive sample preparation. Spectroscopic approaches, such as UV–visible^21,22^ and spectrofluorometric^23–26^ approaches, are operationally simple but often limited by matrix interference. Electrochemical techniques, including potentiometric^27^ and voltammetric methods^28^, offer rapid response, high sensitivity, and environmental compatibility^29–32^, yet their performance can be constrained by slow interfacial electron transfer and limited electrode surface activity, especially in complex samples.

Recent research has emphasized the integration of nanomaterials to overcome these limitations. Carbon quantum dots (CQDs) have attracted considerable interest as effective nanomaterials owing to their nanoscale size, elevated specific surface area, tunable electronic properties, and abundant surface functional groups^33–35^. Biomass-derived CQDs offer a sustainable and cost-effective alternative, combining environmentally friendly synthesis with enhanced electrochemical characteristics^36^. When incorporated into carbon paste electrodes (CPEs), CQDs enhance electron-transfer kinetics, increase active surface sites, and amplify electrochemical signals, thereby improving sensitivity and selectivity^37,38^. In parallel, other nanomaterial-based electrochemical platforms have demonstrated improved analytical performance: epitope-imprinted AuNP-modified electrodes for Prostate-specific antigen detection showed marked selectivity^39^; graphene quantum dots–AuNP composites enabled sensitive Gefitinib quantification with strong anti-interference properties^40^; paper-based electrochemical impedance aptasensors and dual-working screen-printed electrodes provided low-cost, portable Pb^2+^ and Hg^2+^ detection^41,42^; hierarchical mesoporous carbon nanosheets allowed trace-level acetaminophen analysis with rapid proton transfer^43^; and honeycomb-like carbonized Zeolitic Imidazolate Framework-67 aptasensor arrays enabled multiplexed cardiac biomarker detection with high reproducibility and low limits of detection^44^. Collectively, these studies highlight the advantages of nanostructured materials, surface engineering, and aptamer recognition in enhancing electrochemical sensor performance.

Despite these advances, naturally derived CQDs remain scarcely explored for the electrochemical determination of VON in complex matrices. An environmentally benign electrochemical sensor is proposed in this work using pecan nut–derived CQDs integrated into a CPE, designed to overcome slow electron-transfer kinetics and limited electrode surface activity. Unlike previously reported CQD-based sensors relying on synthetic materials or complex fabrication, the present approach employs a biomass-derived platform prepared via rapid microwave synthesis. The resulting defect-rich CQDs enhance electron-transfer kinetics, as confirmed by reduced charge-transfer resistance and improved voltammetric response. Additionally, the sensor was successfully applied to pharmaceutical formulations, plasma, and synthetic wastewater. These features provide a practical, sensitive, and eco-friendly analytical platform for VON determination.

## Experimental

### Materials and reagents

High-purity VON, serving as the reference standard, was obtained from Hikma Pharmaceuticals, Egypt (batch no. 2202110829) and used without further modification. The commercial pharmaceutical formulation, Topoprazan^®^ tablets 20 mg, was procured locally (batch no. 015, Hikma Pharmaceuticals).

The pecan nuts (*Carya illinoinensis*) used for CQDs synthesis were purchased from a local commercial supplier (Abu Auf, Cairo, Egypt).

All solutions were prepared with distilled water. The CPE was fabricated using liquid paraffin (99%, PIOCHEM, Egypt) and graphite powder (extra pure, 98.5%, LANXESS, Germany). Electrodes were further modified with multi-walled carbon nanotubes (MWCNTs, Sigma-Aldrich, Germany), zinc oxide nanoparticles, cadmium oxide nanoparticles (< 50 nm, 99.9% metal purity), and polyethylene glycol 400 (PEG-400, Alpha Chemika, India) which acted as a stabilizer in CQD synthesis. Buffer solutions were prepared using glacial acetic acid (99.8%, PIOCHEM, Egypt), and sodium hydroxide (99.8%), together with boric acid (99.9%), while phosphoric acid (85% purity; obtained from LOBA Chemie, India) was employed to maintain the desired pH and ionic strength. Potassium hexacyanoferrate (II)/(III) (SMART-LAB, Indonesia) served as a standard redox probe for electrochemical characterization. Other high-purity reagents, including salts such as sodium chloride, potassium chloride, potassium hydrogen phosphate, ammonium chloride, and anhydrous sodium sulfate, were employed in the preparation of synthetic industrial wastewater. Other reagents were used without further purification, consisting of 99.8% acetone of HPLC purity supplied by Central Drug House (India), while the LC–MS grade water was purchased from Sigma-Aldrich in Germany. The remaining materials, all at 99% purity, consisted of lactose from PIOCHEM, maize starch from Biotech, and both hydrochloric acid and sodium lauryl sulfate (SLS) (USP grade) from ALFA Chemical.

### Synthesis of carbon quantum dots and fabrication of a modified carbon paste electrode

**Scheme 1:**
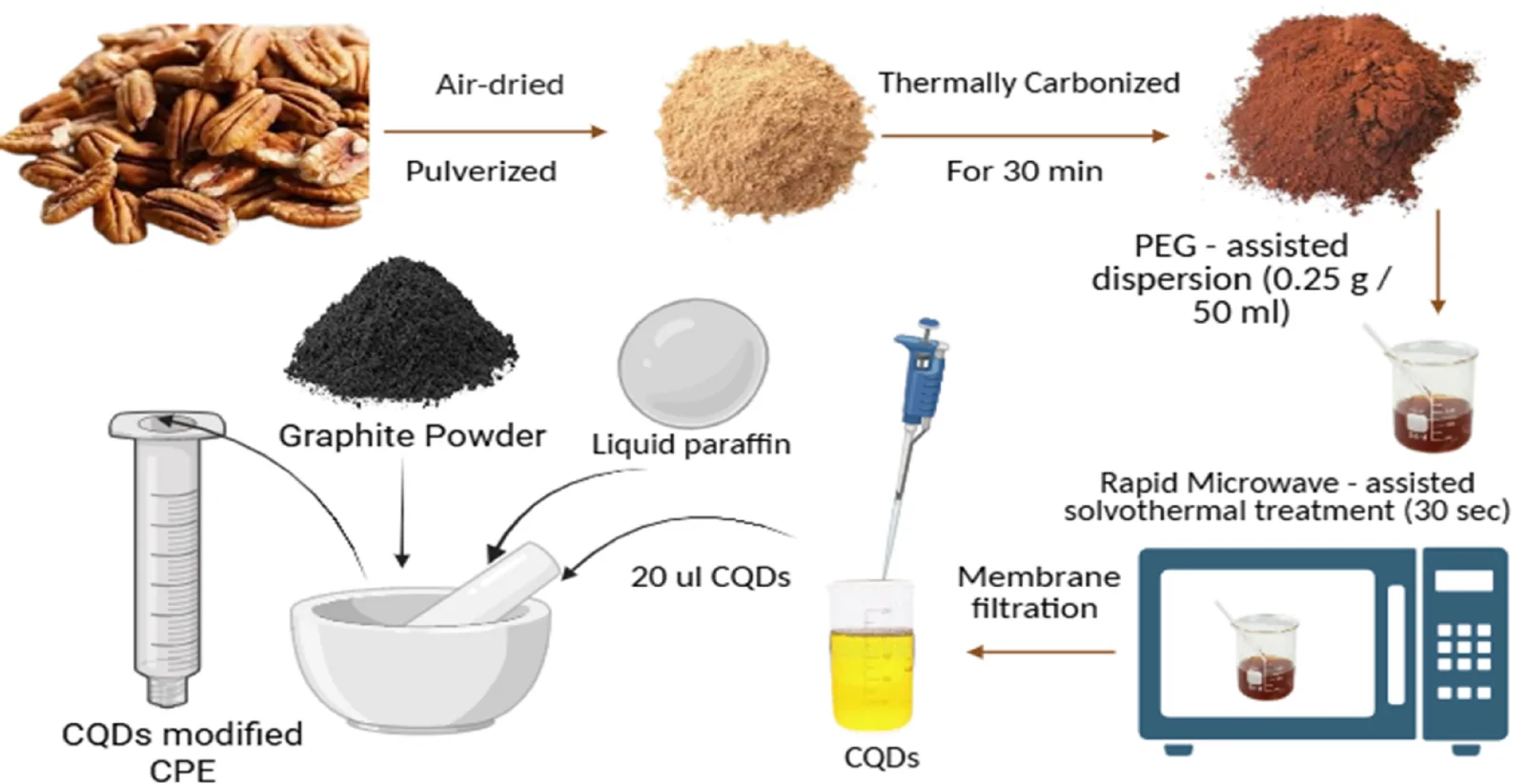
Conceptual schematic delineating the synthetic pathway of CQDs alongside the fabrication strategy for a CQD-functionalized CPE.

Harnessing pecan nut (*Carya illinoinensis*) as a novel and sustainable carbon source, CQDs were synthesized via a microwave-assisted solvothermal synthetic route shown in Scheme 1. Subsequently, the nuts were finely ground into a yellow powder and carbonized at 300 °C for 30 min, yielding a brown carbonaceous residue. PEG 400 (50 mL) provided the liquid phase in which 0.25 g of the material was incorporated for dispersion, stirred to homogeneity, and subjected to microwave irradiation for 30 s under solvothermal conditions, producing a deep brown CQD solution. The resulting dispersion was purified by centrifugation at 6000 rpm for 30 min of processing, the mixture was passed via membrane filters of 0.45 μm and then 0.22 μm pore size in sequence, yielding a transparent and stable CQD solution, which was stored at 4 °C for subsequent applications.

The bare CPE was constructed by intimately homogenizing liquid paraffin with graphite powder at a 0.6 mL : 1 g ratio, adapted from the literature-established 0.3 mL : 0.5 g composition via proportional scale-up^45^, producing a malleable carbonaceous paste. The paste was deposited into an insulin syringe; electrical interfacing was achieved through the incorporation of a metallic wire. The electrode surface was then refined through gentle polishing to produce a reproducible and electrochemically active interface.

A set of nanostructured CPEs was prepared by dispersing 1–2% (w/w) of MWCNTs, ZnO, or CdO into graphite powder, followed by incremental addition of paraffin oil to form uniform, cohesive pastes. For the final CQDs/CPE, 20 µL of the prepared CQDs were incorporated to produce a conductive, stable nanocomposite. Exhibiting enhanced electron-transfer kinetics and electrochemical stability, the CQDs/CPE served as the main sensing electrode, while the remaining nanostructured CPEs provided comparative benchmarks.

### Preparation of standard solutions

For electrochemical investigations, an accurately weighed amount of VON was initially dissolved using a 10.0 mL aliquot of 0.1 N HCl and diluted with distilled water up to the required volume in a 100 mL volumetric flask, yielding a 1.0 × 10^−3^ mol L^−1^ stock solution that served as the reference throughout the study.

### Instrumentation and experimental procedures

All electrochemical experiments were executed using an Origaflex multichannel workstation (Origalys Electrochem SAS, France), interfaced with Origamaster 5 software for control and data logging. The configuration relied on a standard three-electrode assembly: a platinum electrode functioned as the counter, an Ag/AgCl/KCl electrode served as the reference, and the CQDs/CPE was utilized as the working electrode. Acidity of the test solutions was monitored with an Adwa AD1030 pH meter (Romania).

All recorded voltammograms represent raw experimental data, and peak currents (${I}_{p}$) were evaluated directly using the workstation software by measuring the vertical peak height relative to the extrapolated background baseline, without baseline correction or background subtraction.

CQDs were synthesized via microwave-assisted processing using a MAS-II PLUS system (SINEO, China). The precursors were dried, homogenized, and centrifuged (EBA 200, Hettich, Germany) to obtain a stable colloidal suspension.

The CQDs were characterized to evaluate their optical, structural, and surface properties. Edinburgh Instruments (UK) provided the FS5 spectrofluorometer used for fluorescence profiling, while an Acculab UVS-85 device was employed to capture UV–Vis absorption data. A Bruker Alpha II (Germany) was used to map surface functional chemistry via FTIR. Internal morphology, along with size distribution data, was provided by a JEOL JEM-2100 F high-resolution transmission electron microscope. Furthermore, electronic environments and elemental profiles were evaluated on a Thermo Fisher Scientific K-Alpha XPS system (USA). To complement these findings, an EDAX SEM–EDX system (USA) was employed, integrating scanning electron microscopy with energy-dispersive X-ray analysis for comprehensive topographical and elemental mapping.

#### Cyclic voltammetric measurements

The anodic electrochemical behavior of VON was probed using cyclic voltammetry. A linear potential sweep spanning 0 to + 1.5 V was applied at a scan rate of 100 mV s^−1^ with a resolution of 5 mV per step. All procedures were conducted under controlled experimental conditions to ensure reproducible monitoring of the anodic oxidation behavior of VON.

#### Differential pulse voltammetric measurements

The anodic oxidative response of VON was examined by means of differential pulse voltammetry (DPV). The electrochemical response was evaluated by sweeping the applied potential from 0 up to + 1.5 V. Data acquisition was performed using a 5 mV step resolution, alongside a 50 mV modulation amplitude, while maintaining a constant scan rate of 100 mV s^−1^ throughout the measurements. This experimental protocol enabled consistent registration of the anodic oxidation signal of VON at around + 1.1 V. All measurements were carried out under carefully controlled conditions to ensure methodological reliability and measurement repeatability.

### Preparation of pharmaceutical dosage forms

Tablet samples containing VON were sourced from Topoprazan^®^ formulations, each labeled with a nominal content of 20 mg per tablet. Following the optimized DPV parameters, the analytical procedure began by establishing the average mass of a ten-unit sample. These tablets were then ground into a fine, consistent texture using a mortar. To represent the strength of an individual dosage unit, a specific quantity of this powder was introduced into a 100 mL glass volumetric container. Solubilization was promoted by adding HCl (0.1 N) in a 10.0 mL volume, followed by a period of sonication to ensure the VON was entirely released into the liquid phase. Distilled water was then utilized to reach the 100 mL graduation limit. To isolate the clear analyte from non-soluble filler materials, the resulting mixture was processed through a filtration step. Finally, the clarified stock liquid was sampled to prepare the necessary working dilutions for the electrochemical assays.

### Preparation and analysis of spiked human plasma samples

Blank human plasma was selected as a biological matrix to evaluate the applicability of the proposed method. Plasma samples were fortified with VON at three concentrations within the validated linear range. Proteins were precipitated using acetonitrile, followed by vortexing and centrifugation. The recovered supernatant was buffered with 0.04 M BRB solution (pH 9), after which particulate matter was eliminated using a 0.22 μm microfiltration step prior to electrochemical determination by DPV under optimal conditions. Triplicate analyses were carried out to establish method precision and evaluate susceptibility to matrix-induced variability.

### Preparation and analysis of synthetic pharmaceutical wastewater

To assess potential matrix-induced interferences and demonstrate the feasibility of the proposed voltammetric approach for environmental monitoring of VON, a model synthetic pharmaceutical wastewater was prepared. In a 1 L volumetric flask, 0.1 g VON was co-dissolved with 100 ml of 0.1 N HCl, the solution was then fortified with typical interfering species and excipients, including NaCl (0.5 g), $\mathrm{Na}_{2}\mathrm{SO}_{4}$ (0.2 g), starch (0.05 g), $\mathrm{K}_{2}\mathrm{HPO}_{4}$(0.05 g), lactose (0.1 g), $\mathrm{NH}_{4}$Cl (0.05 g), SLS (0.01 g), and acetone (0.5 mL), LC–MS grade water was added to bring the solution to its final volume. The resulting solution was subjected to sonication to achieve complete dissolution and homogeneity, following passage via a 0.22 μm pore-size filtration process for effective clarification and elimination of undissolved impurities, the solution aliquots were adjusted with the supporting electrolyte to mitigate interferences from ionic species and surfactants, bringing VON concentrations within the validated analytical range. DPV was then performed, with each measurement conducted in triplicate to ensure precision, reproducibility, and robustness of the analytical procedure.

## Results and discussion

### Structural and physicochemical evaluation of CQDs and electrochemical behavior of the functionalized electrode

#### Nanoscale morphology and crystallinity

**Fig. 1:**
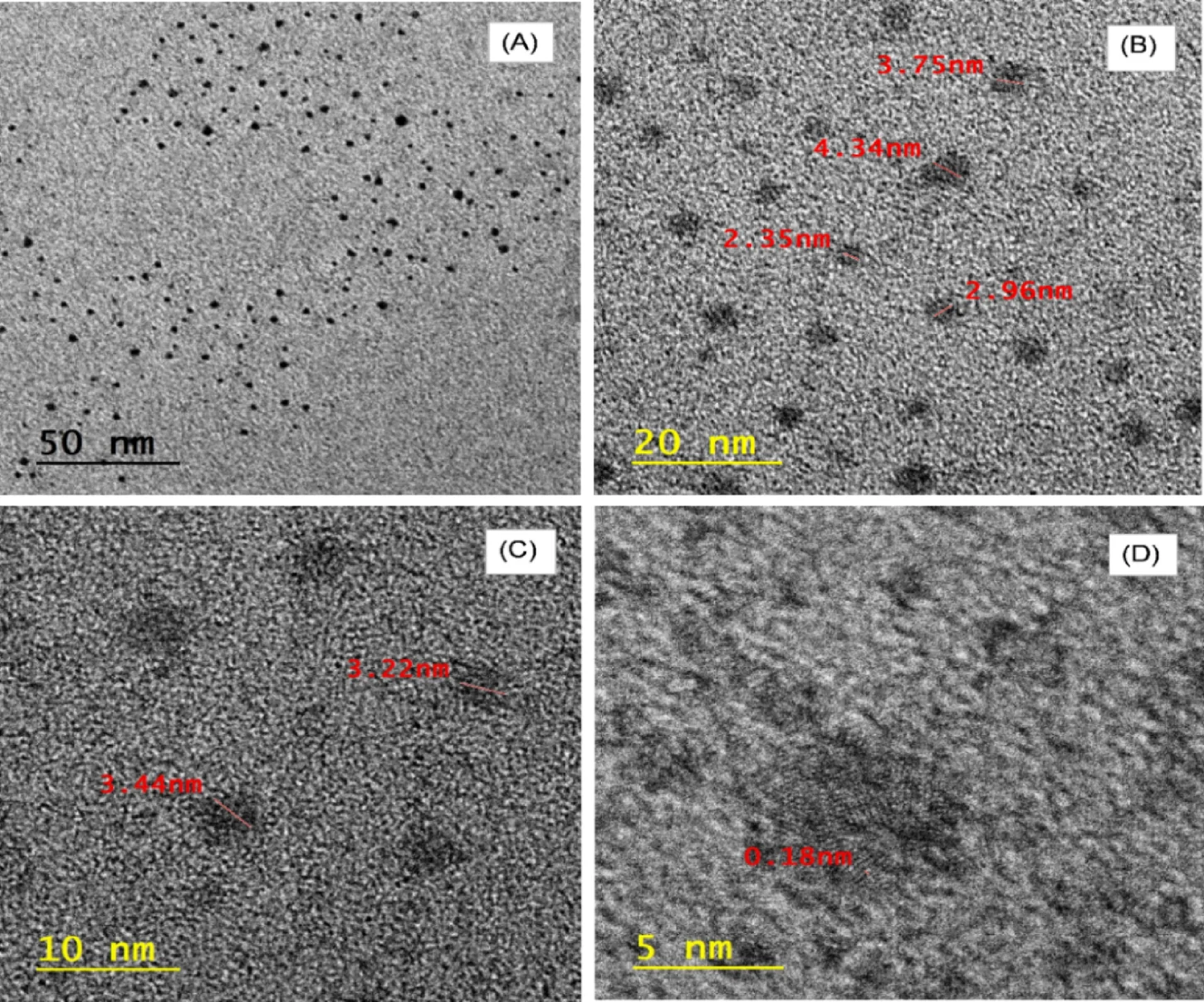
TEM images of CQDs (**A**–**D**) captured at multiple magnifications, illustrating uniform morphology and monodisperse nanoscale particles.

TEM micrographs recorded at different magnifications (Fig. 1) demonstrate the formation of monodisperse, quasi-spherical CQDs with particle diameters of 3.75, 4.34, 2.34, and 2.96 nm, yielding an average size of 3.35 nm. This narrow size distribution confirms controlled nucleation and growth during synthesis^46,47^. HRTEM images further reveal the presence of graphitic carbon domains, as confirmed by distinct lattice fringes displaying an interplanar spacing of 0.18 nm, indicating partial structural ordering within the carbon framework. Such uniform nanoscale morphology provides a consistent electroactive interface, which is essential for reproducible electron-transfer processes^48,49^.

#### Surface chemistry and functional group architecture

The surface chemistry of the CQDs was systematically investigated using FTIR and XPS to elucidate the nature of functional groups and defect-related features. The FTIR spectrum (Fig. S1) exhibits characteristic bands at 3442 cm^−1^ (O–H), 2856 cm^−1^(C–H), 1742 cm^−1^ (C=O), 1632 cm^−1^ (C=C) and 1152–1051 cm^−1^ (C–O), confirming the presence of abundant oxygen-containing functionalities. These groups are known to enhance surface polarity and promote analyte adsorption^50–52^.

XPS analysis (Fig. S2) provides further insight into the surface chemical states. The C 1*s* spectrum reveals contributions from graphitic carbon (C–C/C=C, 284.9 eV), defect-associated carbon species (C–H, 285.6 eV), and oxygenated carbon (C–O, 288.4 eV), indicating partial oxidation and the presence of structural defects. The O 1*s* spectrum confirms C–O/OH and C=O functionalities, supporting the FTIR results. The relative atomic composition obtained from XPS suggests a high density of oxygenated species, which can serve as active sites for interfacial interactions and facilitate charge transfer^53–55^.

#### Optical and electronic structure

The electronic structure of the CQDs was further evaluated using UV–Vis and photoluminescence (PL) spectroscopy. The absorption bands at 325 and 420 nm are attributed to π–π* transitions linked to graphitic domains and n–π* electronic transitions associated with surface states, respectively (Fig. S3A)^56,57^. The strong PL emission centered at 440 nm upon excitation at 310 nm is attributed to defect-mediated radiative recombination (Fig. S3B), confirming the presence of electronically active surface defects. These defect states are expected to introduce localized energy levels that can enhance charge-transfer efficiency^58,59^.

#### Microstructure and elemental composition

SEM analysis (Fig. S4A) reveals aggregated but uniformly distributed nanoparticles, consistent with the TEM observations. EDX results (Fig. S4B) confirm a carbon–oxygen composition (C: 25.29 wt%, 31.08 at%; O: 74.71 wt%, 68.92 at%) with no detectable metallic impurities, verifying the purity of the synthesized CQDs and excluding possible interference from residual catalytic species^60–62^.

#### Interfacial electron-transfer dynamics

**Fig. 2:**
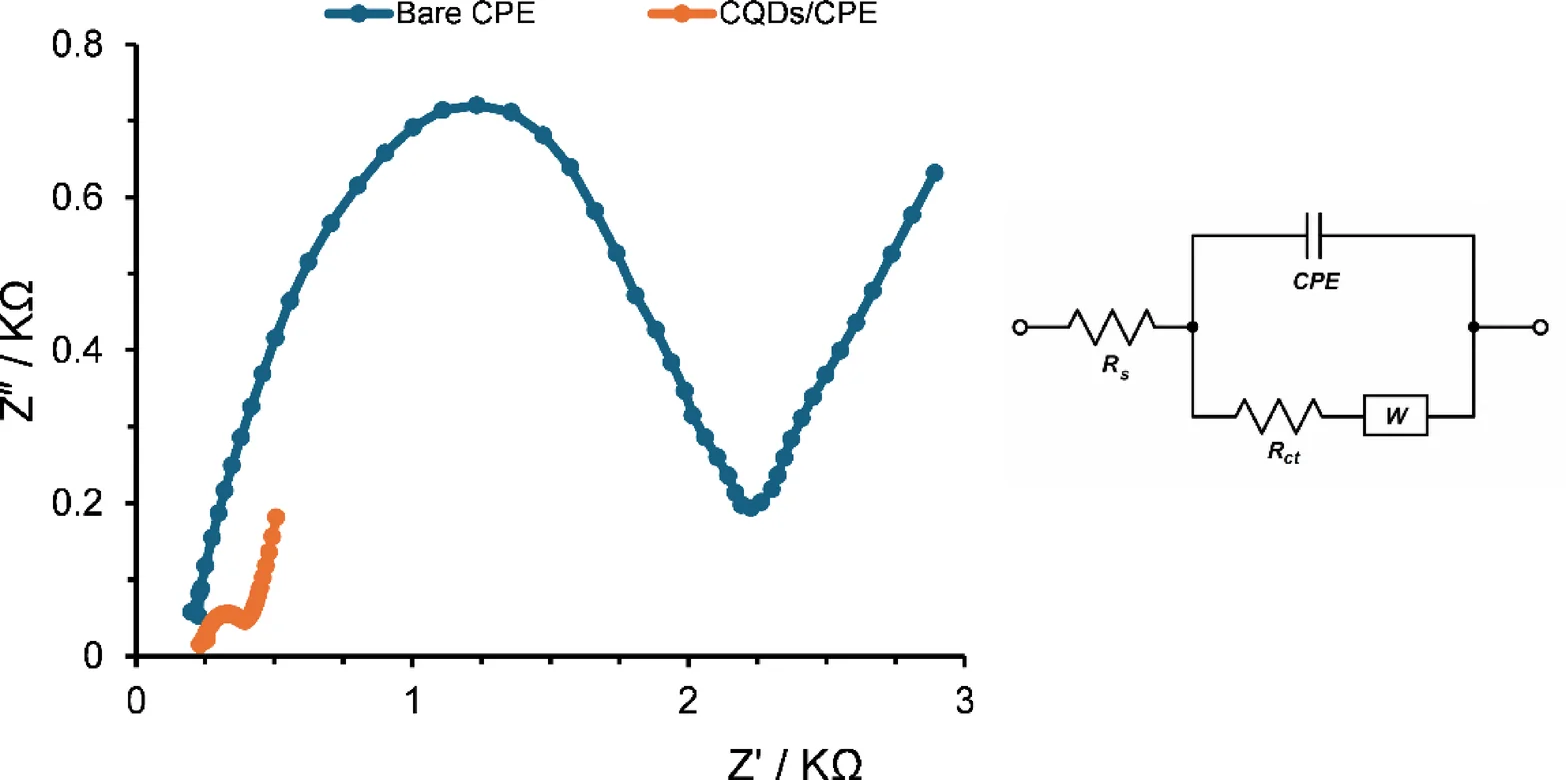
Comparative Nyquist plots of bare CPE and CQDs/CPE, showing substantial reduction in interfacial resistance and improved electrochemical response.

To assess the influence of CQDs’ structural and surface characteristics on interfacial electron transfer, EIS measurements were performed on both the bare CPE and the CQDs-modified electrode (Fig. 2). The Nyquist plots exhibit a high-frequency semicircle and a low-frequency linear region, corresponding to charge-transfer and diffusion-controlled processes, respectively. A pronounced decrease in semicircle diameter was observed after CQD modification, indicating reduced interfacial resistance^63^. The impedance spectra were quantitatively fitted using the modified Randles equivalent circuit [Rs(CPE Rct W)], showing good agreement with experimental data as confirmed by the low χ^2^ values (1.4 × 10^−4^ for bare CPE and 8.2 × 10^−5^ for CQDs/CPE). The fitted Rs values remained nearly constant (0.199 and 0.231 kΩ for bare CPE and CQDs/CPE, respectively), whereas Rct significantly decreased from 2.026 to 0.165 kΩ upon CQD incorporation, demonstrating accelerated electron-transfer kinetics^64^.

The constant phase element parameter (Q) increased from 3.15 to 14.82 µS s^n^, accompanied by an increase in n from 0.82 to 0.91, revealing enhanced interfacial homogeneity and more ideal capacitive behavior. Moreover, the decrease in the Warburg coefficient from 0.385 to 0.124 kΩ s^−1/2^ indicates improved diffusion and mass transport^65^. Collectively, these results confirm that CQD-induced defect sites and oxygenated functionalities enhance interfacial charge transfer, surface homogeneity, and diffusion, thereby improving the electrocatalytic performance of the modified electrode^66^.

### Batch-to-batch consistency and reproducibility assessment of synthesized CQDs

To assess the robustness of the synthesis and address potential variability from biomass precursors, three independent CQD batches (batch 1–3) were evaluated. TEM analysis (Fig. S5) showed mean particle sizes of 2.63, 3.35, and 3.33 nm for batch 1–3, respectively (SD = 0.41), indicating narrow size dispersion. PL measurements (Fig. S6A) exhibited consistent emission behavior across batch 1–3 with minimal intensity variation (SD = 0.87), confirming optical uniformity. XPS analysis (Fig. S6B) demonstrated consistent reproducible surface chemistry across batch 1–3, with atomic percentages of C=C/C–C (71.98, 72.32, 72.09; SD = 0.17), C–H (22.45, 21.73, 22.13; SD = 0.36), and C–O (5.42, 5.59, 5.47; SD = 0.09), alongside consistent oxygen functionalities: C–O/OH (73.25, 73.07, 72.76; SD = 0.25) and C=O (26.75, 26.93, 27.14; SD = 0.19). DPV measurements (Fig. S6C) at 100 nM of VON yielded current responses of 10.27, 11.19, and 10.78 µA for batch 1–3, respectively (SD = 0.46), confirming satisfactory electrochemical reproducibility.

Overall, the minimal variability across batch 1–3 confirms satisfactory synthetic consistency. The absence of mineral or metallic contributions further supports pecan nut biomass as a stable precursor, contributing to reliable sensor performance. The consistency across batches demonstrates that pecan nut biomass provides a reliable source of carbon precursors with reproducible functionalization, supporting the practical scalability of this approach. Such batch-to-batch reproducibility ensures that the electrochemical response remains consistent and reliable across independent syntheses, highlighting the robustness of the CQDs as a sensing material.

### Electrochemical behavior of VON at CQDs-modified carbon paste electrode

**Fig. 3:**
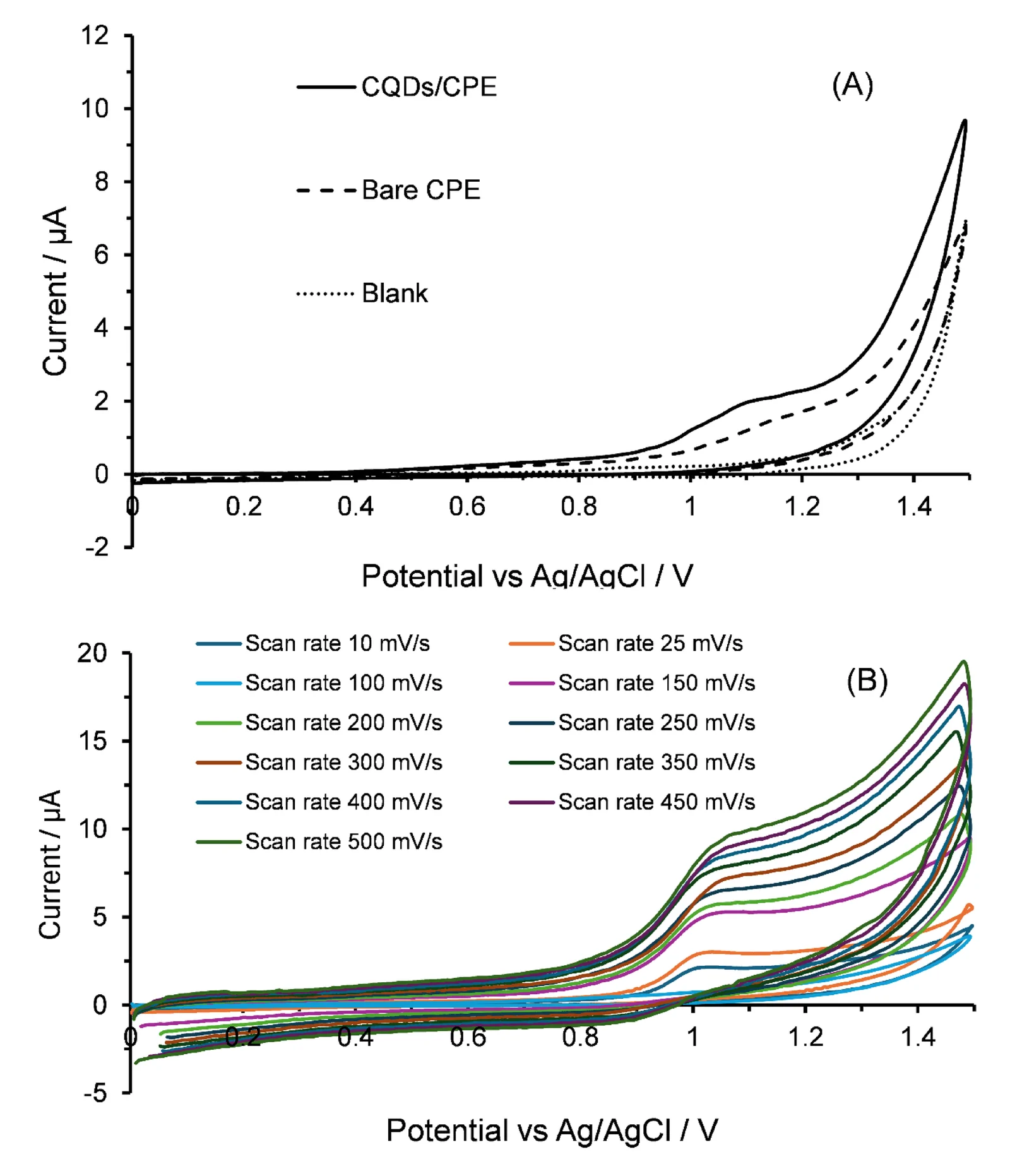
(**A**) Comparative cyclic voltammograms of 50 nM VON recorded at CQDs/CPE, bare CPE, and blank BRB (0.01 M, pH 9), demonstrating the enhanced anodic response of the CQDs-modified interface. (**B**) Scan-rate-dependent cyclic voltammograms of 50 nM VON recorded in 0.01 M Britton–Robinson buffer (pH 9), revealing the kinetic features of the oxidation process.

The voltammetric response of VON was evaluated at a bare CPE and CQDs/CPE in 0.01 M BRB (pH 9) via cyclic voltammetry (0–1.5 V, step potential 5 mV, scan rate 100 mV s^−1^) as shown in Fig. 3A. At the unmodified electrode, VON generated a faint, broad, irreversible anodic peak near 1.1 V, indicative of sluggish electron transfer. Following CQDs modification, a pronounced, sharp peak emerged at the same potential, reflecting substantially accelerated electron-transfer dynamics and amplified electroactive surface accessibility.

This signal intensification is attributed to the conductive carbon network, abundant defect-rich sites, and expanded surface area of the biomass-derived CQDs, which synergistically facilitate rapid electron flow and promote irreversible VON oxidation. Negligible current in the blank buffer confirmed that the observed response originates exclusively from VON. Collectively, these findings highlight the CQDs/CPE as an electrocatalytically enhanced platform for sensitive voltammetric quantification.

The mechanistic pathway of VON electrooxidation was interrogated through scan-rate-dependent cyclic voltammetry across 10–500 mV s^−1^ (Fig. 3B). With increasing scan rate, a progressive increase in the anodic peak current was observed, indicating an increasing contribution of mass transport to the electrooxidation response. To elucidate the controlling mechanism of the oxidation process, the relationship between the logarithm of the anodic peak current (log I_p_) and the logarithm of the scan rate (log ν) was evaluated (Fig. S7A). A strong linear correlation was obtained according to the equation: log I_p_ (µA) = 0.5294 log ν − 1.1043 (R^2^ = 0.9926). The calculated slope value (0.5294) is close to the theoretical value of 0.5 expected for a diffusion-controlled process, while values approaching unity are characteristic of adsorption-controlled behavior^67,68^. Although close to the theoretical value of 0.5, the slope value of 0.5294 was interpreted as part of a multi-parameter kinetic assessment rather than as an isolated criterion. When considered together with the linear I_p_–ν^1/2^ relationship and the Cottrell response, the convergent electrochemical evidence supports a predominantly diffusion-controlled oxidation of VON.

Further confirmation of the mass-transfer-controlled nature of the oxidation process was obtained by examining the dependence of the anodic peak current on the square root of scan rate (${\nu}^{1/2}$) (Fig. S7B). The linear relationship described by ${I}_{p}$ (µA) = $0.1247\,\nu^{1/2}-0.2372$ (R^2^ = 0.9943) demonstrates that the oxidation current follows the characteristic behavior predicted for diffusion-controlled electrochemical reactions^69^. The linearity of the I_p_–ν^1/2^ relationship (R^2^ = 0.9943) provides evidence that the faradaic response is primarily governed by semi-infinite linear diffusion of VON from the bulk solution toward the CQDs/CPE interface, rather than by surface accumulation.

Additionally, chronoamperometric measurements performed at the oxidation potential exhibited a continuous decrease in current with time, consistent with the depletion of electroactive VON species near the electrode surface. The linear correlation between current response and ${t}^{(-1/2)}$(Fig. S7C) expressed as I (µA) = 10.473 ${t}^{(-1/2)}-0.1173$ (R^2^ = 0.9987), agrees well with the Cottrell relationship: I = nFAD^1/2^C/(π^1/2^ t^1/2^)^70^. The linearity of the Cottrell plot provides further evidence for the diffusion-dependent depletion of electroactive VON species within the interfacial diffusion layer.

Collectively, these findings provide mutually supportive evidence for the proposed mass-transfer behavior. The combined evidence from the near-theoretical log I_p_–log ν slope (0.5294), the linear I_p_–ν^1/2^ relationship supporting diffusion from the bulk solution toward the electrode interface (R^2^ = 0.9943), and the Cottrell plot supporting diffusion-dependent depletion (R^2^ = 0.9987) indicates that VON electrooxidation at the CQDs/CPE interface is predominantly a diffusion-controlled process, rather than surface adsorption.

The Randles–Sevcik equation was utilized for estimating the electroactive surface area of the electrodes: Ip = (2.99 × 10^5^) n (αn_0_)^1/2^ A D^1/2^ C ν^1/2^^71^. The bare CPE exhibited 0.004 cm^2^, whereas CQDs/CPE showed 0.008 cm^2^, confirming a two-fold increase upon CQDs modification. This enhancement corroborates the pronounced anodic peak of VON and reflects the improved electron-transfer kinetics and accessibility provided by the defect-rich CQDs network.

### Proposed mechanism of electrochemical oxidation of VON

The oxidation pathway of VON was inferred from the voltammetric characteristics and scan-rate analysis, which collectively indicate an irreversible electrode process. The anodic transformation is attributed to electron abstraction from the terminal amine functionality, generating a reactive intermediate that undergoes proton dissociation to attain structural stabilization^72^.

It is well-established in the literature^73^ that the effect of pH on the peak potential for proton-coupled electron transfer can be rationalized using a generalized relationship:

$${E}_{p}={E}^{0}-\frac{0.059\,y}{n}\,\mathrm{pH}$$

where *y* corresponds to the number of protons involved in the electrochemical step and *n* signifies the number of electrons exchanged.

To rigorously evaluate this relationship, the anodic peak potential was plotted against pH in the range of pH 7.0–12.0, and the corresponding linear regression analysis is presented in Fig. S8A. A linear relationship was obtained, as expressed by the regression equation:

$${E}_{p}\left(\mathrm{V}\right)=1.3792-0.03\,\mathrm{pH}\text{}({R}^{2}=0.9951)$$

The high determination coefficient (R^2^ = 0.9951) confirms the strong linearity and statistical reliability of the data. The experimental slope of − 0.030 V/pH (− 30 mV/pH) closely matches the theoretical Nernstian slope of − 0.0295 V/pH expected for a system with a proton-to-electron ratio (y/n) of 1/2 (i.e., 1 H^+^/2e^−^ transfer). This observation provides quantitative support for the proposed two-electron/one-proton mechanism, substantiating the involvement of protons in the overall oxidation pathway.

To further substantiate the electron-transfer kinetics and validate the proposed mechanism, Laviron’s model for irreversible systems was applied by correlating the peak potential (${E}_{p}$) with the logarithm of the scan rate (log ν)^74^. A well-defined linear relationship was obtained (Fig. S8B), described by ${E}_{p}$= 0.0596 log ν + 0.9806 (R^2^ = 0.9975). Based on the slope and assuming a charge transfer coefficient (α) of 0.5, the number of electrons involved in the rate-determining step was calculated to be 1.99, which is effectively equivalent to a two-electron transfer process.

**Scheme 2:**
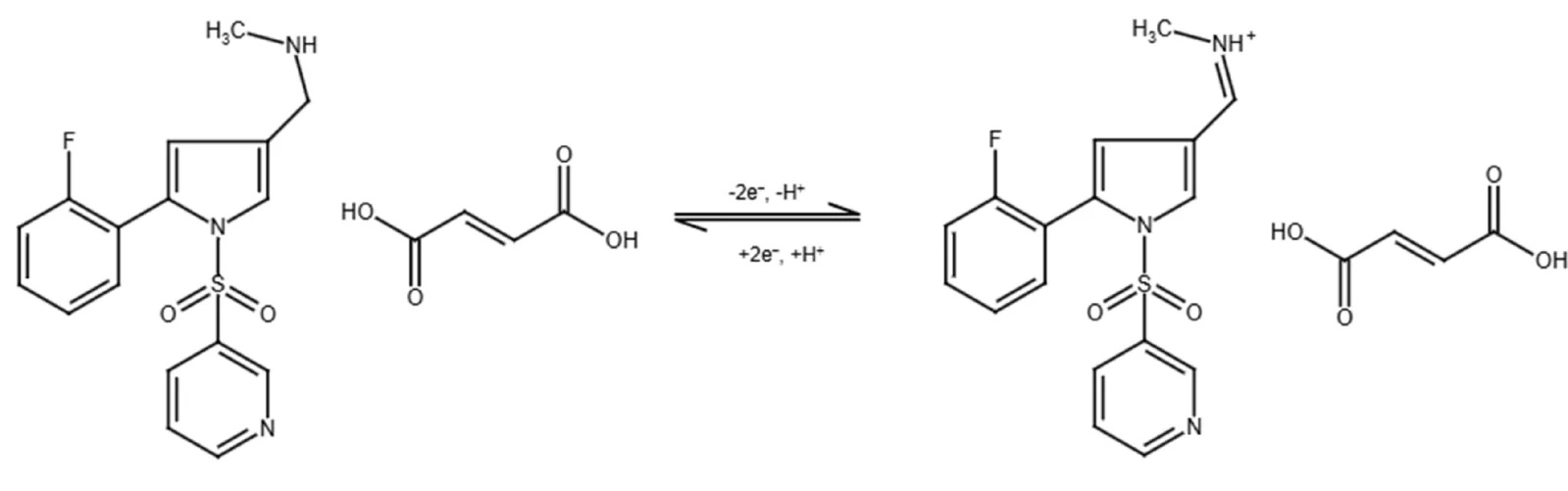
Proposed proton-coupled electron-transfer pathway for the irreversible oxidation of VON.

The overall reaction is therefore governed by a two-electron/one-proton transfer sequence, promoting the conversion of the amine moiety into a more conjugated iminium-like structure accompanied by double-bond development (Scheme 2). This interpretation is further supported by Laviron analysis, which yielded an electron number of (*n* ≈ 2), confirming the involvement of a two-electron transfer step. Combining this with the experimental *E*_*p*_ versus pH slope of − 0.030 V/pH supports the involvement of a single proton (y = 1) in the rate-determining step. The observed response reflects the established anodic behavior of amine-substituted heterocycles, in which proton-coupled electron transfer triggers subsequent chemical evolution that renders the oxidation electrochemically irreversible.

### Voltammetric method optimization and parameter evaluation

Optimization of the electrochemical environment encompassed pH control, CQDs-mediated surface engineering, and adjustment of DPV variables. This integrated tuning markedly improved signal intensity and peak sharpness.

#### Effect of pH and supporting electrolyte

The anodic oxidation of VON was evaluated across pH 2–12 in 0.04 M BRB. In the acidic region (pH 2–6), no oxidation peaks were observed, yielding featureless DPV curves similar to the blank baseline (Fig. S9). This lack of electrochemical activity under acidic conditions may be associated with the protonation of the basic nitrogen moieties of VON. The resulting cationic species may exhibit reduced electron density at the electroactive site, which may contribute to the absence of an observable oxidation signal within the monitored potential window.

Oxidation peaks emerged only in the pH range 7–12. A systematic shift in the peak potential with increasing pH was recorded, indicating that proton availability modulates the rate-determining step, consistent with the proton-coupled electron transfer (PCET) mechanism. Under alkaline conditions, partial deprotonation of the terminal amine enhances its electron-donating capacity, facilitating electron abstraction and stabilizing the intermediate species formed during oxidation.

**Fig. 4:**
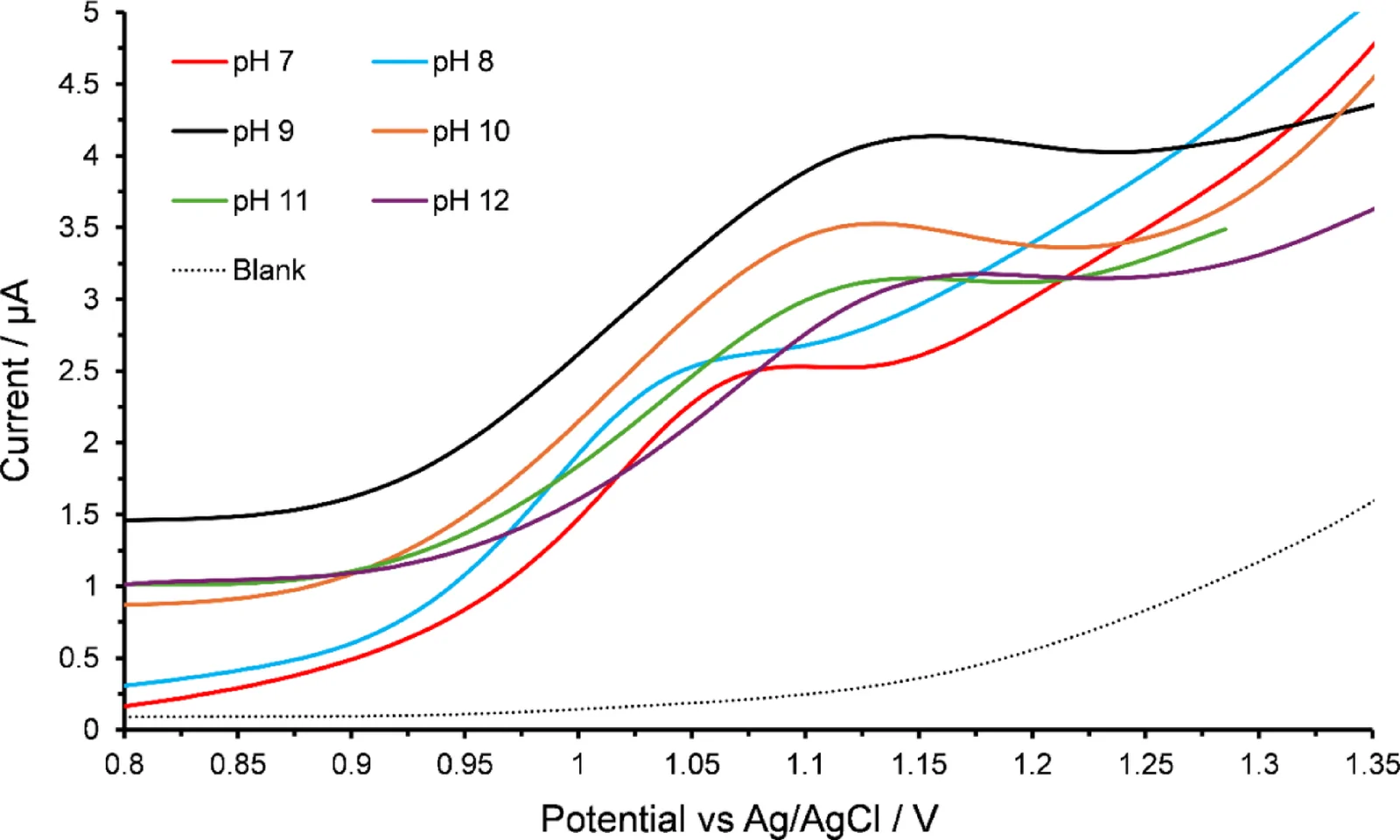
pH-dependent differential pulse voltammetric response of 50 nM VON at CQDs/CPE in 0.04 M BRB, illustrating anodic peak variation (0.8–1.4 V, 5 mV step, 50 mV modulation, 100 mV/s).

The peak current reached a maximum at pH 9 (Fig. 4), reflecting an optimal balance between proton participation and molecular deprotonation. At this pH, the partially deprotonated amine allows efficient charge transfer while retaining sufficient proton involvement for chemical stabilization of the oxidized species. This behavior aligns with scan-rate analysis, which revealed a predominantly diffusion-controlled process. Additionally, favorable interactions between the analyte and oxygen-rich functional groups on the CQDs surface may further enhance electron-transfer kinetics. These mechanistic insights support the two-electron/one-proton oxidation pathway and justify the selection of pH 9 as the optimum medium for sensitive, reproducible voltammetric determination of VON.

In addition, the influence of the supporting electrolyte was examined by comparing 0.05 M borate buffer with 0.04 M BRB at pH 9 (Fig. S10) Although borate buffer provides effective buffering capacity under alkaline conditions, a comparatively lower anodic peak current was obtained relative to BRB. This difference may be attributed to the distinct ionic composition and buffering characteristics of the two systems, which can influence proton-transfer processes and the interfacial charge-transfer behavior at the CQDs-modified electrode surface. In contrast, BRB, as a multicomponent buffer system, provides a suitable ionic environment that facilitates proton exchange and promotes efficient electron-transfer processes. Consequently, 0.04 M BRB was selected as the optimal supporting electrolyte for all subsequent measurements.

#### Effect of electrode surface modification on electrochemical response

**Fig. 5:**
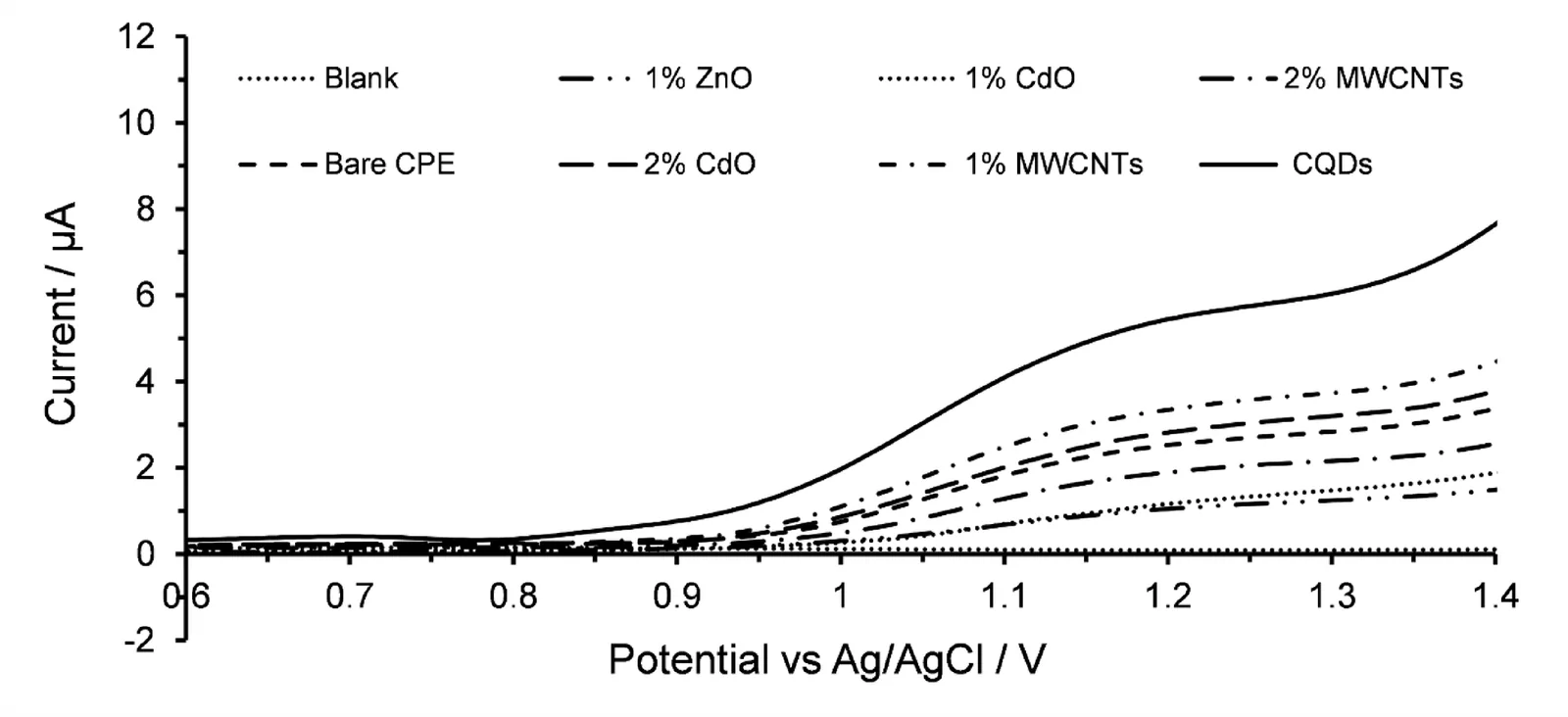
Comparative DPV of 50 nM VON at nanomodified CPEs in 0.04 M BRB (pH 9), demonstrating the critical contribution of electrode interface design to the enhancement of the oxidation signal.

The impact of surface modification on the electrochemical behavior of the electrode was systematically evaluated using MWCNTs (1–2%), CdO (1–2%), ZnO (1%), and CQDs in comparison with bare CPE and the blank, as shown in Fig. 5. The unmodified electrode produced a relatively weak anodic signal, indicating hindered electron-transfer kinetics. Although MWCNTs improved the current response through enhanced conductivity and surface area, CdO and ZnO yielded only modest amplification, likely due to less favorable interfacial charge transport.

Strikingly, the CQDs/CPE exhibited the highest peak intensity, reflecting markedly accelerated charge-transfer kinetics and enhanced analyte accumulation, attributable to the defect-rich carbon matrix and abundant surface functionalities. To assess the potential contribution of PEG-400, blank experiments were performed using CQDs/CPE in the absence of VON. No faradaic response was observed in either CV (Fig. S11A) or DPV (Fig. S11B), confirming that PEG-400 does not contribute to the electrocatalytic enhancement, but rather acts as a stabilizing agent during CQD synthesis; the observed signal arises exclusively from the interaction of VON with the engineered CQD-modified electrode interface. Accordingly, CQDs were selected as the optimal modifier for constructing a sensitive electrochemical sensing platform.

#### Optimization of CQDs interface loading volume

To maximize electrocatalytic efficiency, the loading volume of the biomass-derived CQDs suspension within the carbon paste matrix was systematically varied from 5 to 40 µL. The quantity of this nanostructured modifier crucially dictates the electrochemically active surface area and subsequent charge-transfer resistance. As illustrated in Fig. S12, incremental immobilization of the CQDs solution up to 20 µL induced a progressive amplification in the anodic peak current of VON, attributed to an increased density of graphitic domains and oxygenated surface functionalities that expand accessible electronic surface states and accelerate interfacial electron-transfer kinetics. Conversely, increasing the modifier loading beyond 20 µL resulted in a decrease in the voltammetric response and an increase in the capacitive background current. This performance deterioration is ascribed to excessive accumulation of the carbon nanomaterial, which induces mass-transport limitations, impedes analyte diffusion, and generates a thick, poorly conductive interfacial layer that partially passivates the underlying conductive graphite networks. Consequently, a 20 µL loading volume was established as the optimum operational standard, ensuring an ideal compromise between rapid interfacial kinetics, structural stability, and peak sensitivity.

#### Optimization of differential pulse voltammetric parameters

The electroanalytical efficiency of the fabricated sensing interface is intrinsically governed by instrumental parameters; therefore, key DPV variables were carefully optimized to enhance current response without compromising peak resolution.

#### Scan rate

The DPV scan rate was investigated across 5–120 mV s^−1^ to elucidate its influence on the voltammetric behavior (Fig. S13A). The anodic current increased consistently with scan rate, suggesting facilitated charge-transfer processes and improved electrochemical responsiveness. The signal attained its highest intensity at 100 mV s^−1^; further increments offered negligible enhancement and may promote non-faradaic contributions that adversely affect peak clarity. Consequently, 100 mV s^−1^ was adopted for subsequent analyses as it provides an optimal compromise between signal amplification and voltammetric resolution.

#### Potential amplitude

The impact of pulse amplitude was evaluated over the range of 10–100 mV (Fig. S13B). Elevating the amplitude progressively intensified the oxidation signal up to 50 mV, indicative of strengthened faradaic processes. However, amplitudes beyond this threshold led to noticeable peak widening without a commensurate rise in current, implying diminished analytical precision. Therefore, 50 mV was selected as the optimal pulse amplitude, as it ensures enhanced sensitivity while preserving distinct and well-defined peak morphology.

### Analytical performance of the proposed method

The sensing efficiency of the CQDs/CPE nanosensor for the voltammetric determination of VON was comprehensively evaluated under the optimized experimental conditions. The engineered sensing interface produced sharp, well-resolved oxidation peaks that increased systematically with rising analyte concentration, signifying the strong electrocatalytic influence of the CQDs and the facilitated charge-transfer kinetics at the electrode surface.

**Fig. 6:**
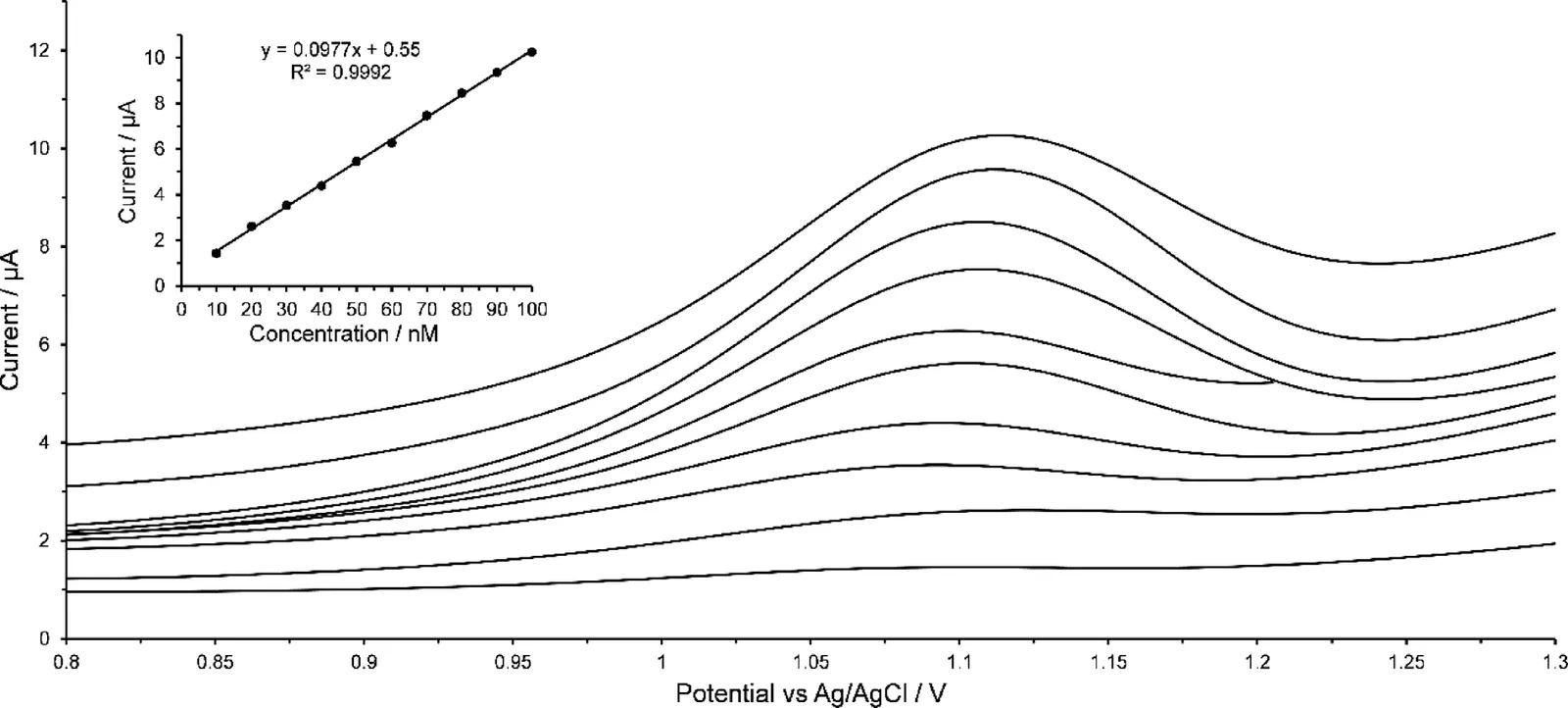
DPV recorded at the CQDs/CPE for successive additions of VON (1.0 × 10^−8^–1.0 × 10^−7^ mol L^−1^) in 0.04 M BRB (pH 9).

A clear linear correlation between the anodic peak current (*I*_*p*_) and VON concentration was established over the ultralow range of 1.0 × 10^−8^–1.0 × 10^−7^ mol L^−1^, as illustrated in Fig. 6. Each concentration was measured in triplicate, and the standard deviation (SD) of the resulting currents was calculated. Error bars representing ± SD were incorporated into the calibration plot to reflect experimental reproducibility. The calibration plot obeyed the regression equation: ${I}_{p}=0.0977\,conc\,\left(n\mathrm{mol}\,{L}^{-1}\right)+0.55$, with a strong correlation coefficient (R^2^ = 0.9992). The slope and intercept of the calibration curve were further validated by their 95% confidence intervals, calculated as 0.0977 ± 0.00227 (0.0954–0.0999) and 0.55 ± 0.1407 (0.409–0.691), respectively, indicating minimal statistical dispersion and reliability of the regression parameters. Residuals, calculated as the difference between observed and predicted anodic peak currents, exhibited a mean of − 0.0015 µA and SD of 0.07993 µA, with random distribution around zero (Fig. S14), confirming linearity and homoscedasticity of the calibration model.

The detection sensitivity was further quantified through determination of the detection and quantification limits (LOD and LOQ), as determined from the blank signal standard deviation (σ) and the calibration curve slope (s), according to accepted validation guidelines (LOD = 3.3 σ/s and LOQ = 10 σ/s). The CQDs/CPE sensor delivered an exceptionally low LOD of 3.9 × 10^−10^ mol L^−1^ and an LOQ of 1.19 × 10^−9^ mol L^−1^, underscoring its effective performance in sub-nanomolar detection. Such enhanced sensitivity can be primarily ascribed to the enlarged electroactive surface area, high density of edge-plane-like sites, and defect-rich carbon structure of the CQDs.

Overall, the linear dynamic range, favorable regression characteristics, and low detection limits support the analytical performance of the proposed sensor for VON quantification in pharmaceutical analysis.

### Method validation

The voltammetric approach was rigorously validated to verify its reliability for VON quantification. Critical performance attributes—accuracy, precision, robustness, stability, selectivity, and interference resistance—were evaluated following recognized validation principles, ensuring consistent analytical performance and methodological integrity.

#### Accuracy

The trueness of the proposed electrochemical method was critically examined to establish its capability for unbiased VON determination. Accuracy was assessed through the analysis of standard solutions at three concentration levels (15, 45, 85 nM) under the optimized experimental conditions (Fig. S15A). Overlaid DPV curves recorded in triplicate for each concentration demonstrated consistent peak current responses and minimal peak potential shift. The calculated recovery values exhibited close concordance with the corresponding theoretical amounts, reflecting minimal systematic deviation and a high degree of analytical exactness. As presented in Table 1, the favorable recovery outcomes substantiate the methodological reliability of the CQDs/CPE sensing platform and confirm its competence for precise quantitative analysis within the investigated range.

**Table 1:** Analytical accuracy of the CQDs/CPE method for VON quantification.

	Conc (nM)	Mean^*^ concentration found (nM)	% Recovery
	15	14.89	99.27
	45	45.21	100.47
	85	84.91	99.89
Mean % recovery			99.88
± SD			0.49
% RSD			0.49

^*^Average of three determinations.

#### Precision

The repeatability and intermediate precision of the CQDs/CPE voltammetric platform were assessed through intraday and interday analyses of standard VON solutions at three concentration levels (20, 50, 80 nM). Intraday precision was evaluated by performing triplicate measurements within a single day (Fig. S15B), whereas interday precision was evaluated across three consecutive days (Fig. S15C). The recorded DPV curves confirmed good signal reproducibility and consistent peak potentials across all replicate trials. Peak current responses were employed to compute relative standard deviations (RSD%), which remained low (ranging from 0.54 to 0.64% intraday and 0.48–0.77% for interday), as summarized in Table 2. These results indicate acceptable reproducibility and operational stability for routine measurements.

**Table 2:** Precision assessment of VON determination using CQDs/CPE (intraday and interday).

	Conc. (nM)	% Recovery	Mean % recovery	SD	% RSD
Intraday precision	20	100.45	99.73	0.6	0.6
		99.76			
		98.98			
	50	99.23	99.79	0.54	0.54
		100.52			
		99.61			
	80	98.86	99.58	0.64	0.64
		99.45			
		100.42			
Inter-day precision	20	101.03	100.35	0.77	0.77
		99.28			
		100.74			
	50	98.63	99.3	0.48	0.48
		99.71			
		99.57			
	80	100.72	99.94	0.55	0.55
		99.62			
		99.49			

#### Robustness

The reliability of the CQDs/CPE voltammetric procedure was systematically examined through the introduction of intentional, minor modifications in operational parameters, including step potential, pulse amplitude, scan rate, and supporting electrolyte pH. The anodic peak currents of VON remained largely unaffected (Table S1), exhibiting only minor fluctuations, which indicates the method’s resilience to typical experimental perturbations. These results highlight the sensor’s operational stability and reliability, confirming that its analytical performance is maintained under subtle deviations in both instrumental settings and solution conditions.

#### Stability and reusability

The performance of the CQDs/CPE electrode and VON solutions was systematically evaluated for both short- and long-term stability. Short-term stability was demonstrated by 10 consecutive DPV scans, retaining > 98% of the initial peak current (RSD = 1.5%, Fig. S16A), accompanied by the corresponding overlaid voltammograms showing consistent peak shape, potential, and current response (Fig. S16B), indicating negligible surface fouling and good signal reproducibility.

Long-term stability over 14 days under refrigerated conditions preserved > 95% of the initial response (Fig. S16C), while ambient storage showed a moderate decline (~ 88%, Fig. S16C), supported by representative DPV curves confirming signal behavior over time under both conditions (Fig. S16D), indicating better signal retention under low-temperature storage.

Reusability was assessed over 20 successive measurement cycles, and the relative peak current retention was calculated for each cycle, with < 5% overall signal loss (Fig. S16E), alongside the corresponding overlaid DPV profiles (Fig. S16F), while independent electrode preparations exhibited an RSD of 2% (Fig. S16G), with overlaid voltammograms directly demonstrating consistent peak intensity and potential across independently fabricated electrodes (Fig. S16H), supporting reproducibility of electrode fabrication. Collectively, these results demonstrate a consistent electrochemical response under the investigated conditions.

#### Selectivity and interference evaluation

The selectivity of the CQDs/CPE voltammetric platform toward VON was systematically evaluated both individually and under simultaneous conditions in a mixed solution containing co-administered or substituted drugs, structurally related acid-suppressing agents, antibiotics used for *H. pylori* eradication, common electroactive species, and pharmaceutical excipients. The study included proton pump inhibitors such as esomeprazole, antibiotics including amoxicillin and levofloxacin, representative electroactive biomolecules such as ascorbic acid and uric acid, and excipients comprising croscarmellose sodium, corn starch, magnesium stearate, and lactose monohydrate.

DPV measurements obtained for the multicomponent mixture (Fig. S17A) as well as individual binary solutions containing VON with each specific interferent (Fig. S17B–F) showed that these species induced negligible changes in the anodic peak current of VON, with no observable overlapping signals within the studied potential window. The anodic peak of VON appeared at approximately 1.10 V, distinctly separated from the oxidation potentials of potential interferents—amoxicillin (~ 0.30 V)^75^, uric acid (~ 0.20 V)^76^, ascorbic acid (~ 0.50 V)^77^, esomeprazole (~ 0.70 V)^78^, and levofloxacin (~ 0.80 V)^79^—indicating clear peak separation under the investigated conditions.

Furthermore, quantitative assessment of the relative peak current response (%) of VON in the presence of individual interferents, excipients, and the combined mixture confirmed minimal signal variation (< 5% relative error, *n* = 3), as depicted in the corresponding bar chart with error bars (Fig. S17G). These results indicate that the tested co-existing species had a limited effect on the VON response under the investigated conditions. Accordingly, the proposed sensor can be applied for the quantification of VON in the investigated pharmaceutical and biological matrices.

### Investigation of method performance in pharmaceutical formulations, biological fluids, and environmental samples

The performance of the CQDs/CPE voltammetric method was evaluated across pharmaceutical formulations, biological matrices, and environmental samples. For commercial dosage forms, the method accurately quantified VON (Table S2), confirming its suitability for routine quality control. In human plasma samples spiked with VON, recoveries and precision metrics (Table S3) validated its bioanalytical applicability. Finally, synthetic industrial wastewater was analyzed (Table S4), demonstrating reliable performance in complex aqueous matrices. Collectively, these studies highlight the method’s broad scope, accuracy, and reproducibility, establishing it as a reliable tool for pharmaceutical, clinical, and environmental analyses.

### Comparative evaluation of the developed analytical method

Chromatographic methodologies^14–19^ are fundamentally governed by reversed-phase interactions on C18 stationary phases coupled with organic mobile phases, enabling precise multi-analyte quantification and impurity profiling via hydrophobic partitioning equilibria. UHPLC^20^ extends this paradigm by integrating high-pressure operation, gradient elution schemes, and structurally selective stationary phases (e.g., phenyl-hexyl), thereby improving separation efficiency and resolution. Despite these advantages, such techniques remain dependent on instrumentally intensive platforms and solvent-demanding protocols.

UV spectrophotometric approaches^21,22^ rely on aqueous media or oxidative derivatization pathways (e.g., NBS-mediated reactions), where detection is based on electronic absorption or chromophore formation. Although operationally simple, their reliance on optical signal transduction inherently restricts selectivity, particularly in complex matrices. In contrast, spectrofluorometric methods^23–26^ offer enhanced sensitivity through fluorescence-based mechanisms, including quenching interactions, dye-assisted complexation, and CQDs-enabled emission systems. However, these approaches frequently necessitate derivatization steps or carefully engineered probe environments, which may limit practical applicability.

Electrochemical techniques provide a more direct and sensitive analytical interface. Potentiometric sensors^27^ generate responses through phase-boundary potentials consistent with Nernstian behavior. Previously reported voltammetric systems^28^ utilize nanostructured composites such as Ce-doped ZnO/rGO synthesized via hydrothermal routes, where signal generation arises from surface-confined electrocatalytic oxidation processes, facilitated by enhanced conductivity, enlarged active surface area, and abundant redox-active sites. Nevertheless, these systems often involve multi-step fabrication procedures and reliance on inorganic nanomaterials.

In contrast, the CQDs/CPE sensor developed herein is fabricated via a facile one-pot synthesis of biomass-derived carbon quantum dots, offering a sustainable and reproducible platform. The sensing mechanism is associated with defect-mediated electron-transfer kinetics, wherein oxygen-containing functional groups and intrinsic structural defects introduce localized electronic states that enhance charge transport and promote interactions with VON, thereby amplifying the electrocatalytic response. Compared to existing electrochemical systems, the CQDs/CPE sensor combines sustainable synthesis, defect-rich electron-transfer enhancement, and broad applicability, representing a clear original contribution.

As evidenced in Table 3, these combined attributes not only advance mechanistic understanding but also translate into reliable analytical performance for VON determination in pharmaceutical, biological, and environmental matrices.

**Table 3:** Comparison of the proposed voltammetric method for VON with reported literature in terms of sensitivity and linear range.

Method	Linear range (µg/mL)	LOD (µg/mL)	LOQ (µg/mL)	*R* ^2^	Reference
HPLC Dosage forms and synthetic gastric juice	1-100	0.24	0.80	0.9999	^14^
HPLC Impurities	0.299–29.885	0.090	0.299	0.9988	^16^
RP-HPLC Dosage form	7.5–22.5	-	-	0.9999	^17^
RP-HPLC Dosage form	4–40	0.182	0.552	0.99996	^18^
UHPLC Impurities	0.03–0.75	-	-	0.9999	^20^
UV Dosage form	2–30	0.24	0.70	0.9998	^21^
UV Dosage form	1.0–14	0.29	0.97	0.9990	^22^
Spectrofluorometry Dosage form	0.05–3.00	0.01097	0.03323	0.9994	^23^
Spectrofluorometry Human plasma	0.015–0.20	0.0028	0.0085	0.9998	^24^
Spectrofluorometry Dosage form and Human plasma	0.01–0.40	0.0012	0.0036	0.9997	^25^
Spectrofluorometry Dosage form	4.6–36.8	0.99	3.02	0.999	^26^
Potentiometry Dosage form and Human plasma	0.00399–3994	0.00123	-	0.9998	^27^
Voltammetry Urine and Blood samples	0.0799–3.195	0.00719	0.0024	0.9903	^28^
Voltammetry Dosage form, Human plasma, synthetic industrial wastewater	0.00399–0.03994	0.000156	0.0004753	0.9992	This work

### Evaluation of method greenness and environmental compatibility

Amid the shift toward sustainable laboratory practices, the ecological attributes of the proposed methodology were evaluated. The assessment emphasized reducing hazardous reagents, minimizing solvent consumption and waste, and streamlining procedures while preserving analytical efficiency. The proposed methodology provides a sustainable alternative without sacrificing sensitivity, precision, or robustness.

To ensure thorough and multidimensional appraisal, the sustainability performance of the proposed procedure was evaluated using five established analytical metrics. For comparative assessment, a reported RP-HPLC method^18^ was selected as the reference benchmark. RP-HPLC represents a widely established and routinely applied analytical technique for pharmaceutical quality control and the determination of VON. However, chromatographic methods typically involve the use of considerable volumes of organic solvents and generate chemical waste. Therefore, comparison with this representative chromatographic approach provides a relevant basis for evaluating the environmental and practical advantages of the proposed voltammetric method.

#### Analytical eco-scale

The environmental friendliness of the designed voltammetric method was evaluated using the Analytical Eco-Scale, a semi-quantitative metric assigning penalty points for reagent toxicity, solvent consumption, energy use, and waste generation to yield an overall sustainability score. Methods scoring above 75 are considered green^80^. The proposed strategy achieved a favorable score of 90, reflecting its minimal chemical hazards, conservative solvent usage, and low waste generation. By comparison, the reported chromatographic method^18^ scored 83, indicating reasonable greenness but a relatively greater ecological burden (Table 4). These findings emphasize the lower environmental footprint of the voltammetric approach and support its adoption as a more sustainable analytical alternative.

#### AGREE (analytical greenness metric) approach

A comprehensive and visually informative evaluation of analytical procedures is achieved through the AGREE (Analytical GREEnness) metric, which integrates all 12 guidelines of green analytical chemistry into a single score spanning 0 (least green) to 1 (most green)^81^. The proposed voltammetric method attained a high score of 0.83, reflecting its reduced chemical hazards, minimal solvent consumption, and streamlined operational workflow. In contrast, the reported chromatographic method^18^ scored 0.56, indicating moderate greenness and a comparatively higher environmental burden. These findings, summarized in Table 4, underscore the enhanced sustainability of the voltammetric approach and its suitability as a greener analytical alternative.

#### Complex GAPI (complementary green analytical procedure index)

The Complex GAPI framework offers a multidimensional, color-coded visualization of an analytical method’s environmental performance, capturing the ecological impact across diverse procedural and chemical parameters^82^. The proposed voltammetric method demonstrated a favorable profile, with 1 red, 3 yellow, and 11 green zones, highlighting its minimal ecological footprint and strong alignment with the principles of green analytical chemistry. In contrast, the reported chromatographic method^18^ presented 3 red, 8 yellow, and 4 green zones, indicating a comparatively greater environmental burden (Table 4). These findings underscore the enhanced sustainability of the voltammetric strategy and further substantiate its role as a more environmentally responsible analytical alternative.

#### BAGI (blue applicability grade index) approach

The BAGI provides an integrated evaluation of analytical methods by combining ecological performance with operational practicality, yielding a unified metric that captures both sustainability and functional efficiency^83^. The proposed voltammetric method attained a strong score of 75, reflecting an optimal balance between environmental friendliness and analytical effectiveness. In contrast, the reported chromatographic method^18^ scored 65, indicating moderate greenness accompanied by greater procedural and resource demands (Table 4). These results further highlight the favorable environmental and practical profile of the voltammetric strategy, reinforcing its suitability as a greener and more efficient analytical alternative.

#### White analytical chemistry (RGB12) assessment

This approach offers a comprehensive evaluation by integrating three core dimensions: environmental (green), analytical performance (red), and practical feasibility (blue). It generates a single composite score reflecting the overall sustainability, efficiency, and applicability of the method^84^. Applying this metric, the proposed voltammetric method achieved a score of 95.9, demonstrating alignment with green chemistry principles alongside good analytical performance and practical applicability. In contrast, the reported chromatographic method^18^ scored 80.9, reflecting good overall performance but a comparatively higher environmental and procedural burden (Table 4). These results highlight the enhanced eco-analytical balance of the voltammetric strategy, reinforcing its suitability as a sustainable and efficient analytical platform.

**Table 4:** Green metrics-based comparison between the proposed analytical methodology and an established chromatographic method reported in the literature.

Analytical Eco-Scale	The reported method^18^		The proposed method	
		Penalty points		Penalty points
Reagents	Sodium hydroxide	2	0.01 N HCl	0
	Acetonitrile	4	0.04 M BR buffer	2
	Potassium dihydrogen phosphate	2	Liquid Paraffin	2
	Sodium dihydrogen phosphate	2	Graphite powder	1
			PEG (400)	0
Energy consumption		2		0
Occupational hazard		0		0
Waste		5		5
Total penalty points		17		10
Score	83 (excellent green analysis)		90 (excellent green analysis)	
AGREE				
Complex GAPI				
BAGI				
RGB12				

## Conclusions

The present study introduces a green voltammetric sensor for the sensitive determination of VON, distinguished by the incorporation of CQDs synthesized from pecan nut (*Carya illinoinensis*) as a renewable, natural-source nanomaterial. This sustainable nanomaterial not only significantly amplifies interfacial electron-transfer kinetics and electrochemical responsiveness but also embodies a green analytical strategy by minimizing hazardous reagents, reducing solvent usage, and lowering overall environmental impact. The sensor exhibited satisfactory analytical precision, robustness, and operational simplicity, and its successful deployment in pharmaceutical preparations, plasma samples spiked with analyte, and simulated industrial effluents highlights its versatility and practical relevance. This work goes beyond conventional CQD-based sensing by integrating sustainable synthesis, defect-engineered electrochemical enhancement, and validated real-sample applicability into a single platform. By merging biomass-derived nanomaterials with advanced electroanalytical methodology, this work provides a promising and environmentally responsible sensing platform, offering a potential avenue for the development of next-generation green sensors in pharmaceutical and environmental monitoring.

## Supplementary Information

Below is the link to the electronic supplementary material.

Supplementary Material 1

## Data Availability

Data will be made available on request.
