# Supplementary material for: Pecan nut–derived carbon quantum dots–modified electrode for sensitive electrochemical determination of vonoprazan using a green analytical approach
Source: Sci Rep. 2026 Sep 8;16:28064. doi: 10.1038/s41598-026-68500-y (PMC13550488; doi:10.1038/s41598-026-68500-y)
Supplement: Supplementary file 1 [file 41598_2026_68500_MOESM1_ESM.docx]

**Pecan nut–derived carbon quantum dots–modified electrode for sensitive electrochemical determination of vonoprazan using a green analytical approach**

Arwa Gamal Elbanna ^a,*^, Aya Barseem ^a,b^, Hytham M. Ahmed ^a,b,**^

*^a^ Pharmaceutical Analysis Department, Faculty of Pharmacy, Menoufia University, Egypt*

*^b^ Department of Pharmaceutical Analytical Chemistry, Faculty of Pharmacy, Menoufia National University, 70 Km Cairo-Alexandria Agricultural Road, Menoufia, Egypt*

*Corresponding author

**Corresponding author

E-mail addresses: [Arwa.Gamal88@phrm.menofia.edu.eg](mailto:Arwa.Gamal88@phrm.menofia.edu.eg) (Arwa Gamal Elbanna),

E-mail addresses: [hmaahmed@yahoo.co.uk](mailto:hmaahmed@yahoo.co.uk) (Hytham M. Ahmed).

**Fig. S1.** FTIR spectrum confirming functional group distribution on the CQD surface.

**Fig. S2.** X-ray photoelectron spectroscopy (XPS) analysis of CQDs showing (A) elemental survey and deconvoluted (B) C 1s and (C) O 1s spectra.

**Fig. S3.** Spectroscopic characterization of CQDs: (A) UV–Vis absorption spectrum highlighting their electronic absorption features, and (B) Photoluminescence (PL) emission spectrum recorded under 310 nm excitation wavelength, featuring peak emission centered at 440 nm.

**Fig. S4.** Surface morphology and elemental analysis of CQDs: (A) SEM image depicting aggregated yet uniformly distributed nanoparticles, and (B) EDX spectrum confirming elemental carbon and oxygen (C/O) composition with no detectable metallic impurities.

**Fig. S5.** TEM images (A-C) showing particle size distribution of CQDs for batches 1–3.

**Fig. S6.** Batch-to-batch reproducibility assessment of CQDs across three independent synthesis batches (Batches 1–3): (A) Photoluminescence (PL) spectra showing consistent optical emission behavior; (B) XPS spectra confirming reproducible surface elemental composition; and (C) Overlaid DPV responses recorded at the CQDs/CPE interface for 100 nM VON, demonstrating satisfactory electrochemical reproducibility.

**Fig. S7.** Kinetic and mass transport characterization of VON electrooxidation at the CQDs/CPE interface: (A) Double logarithmic plot of log peak current versus log scan rate, confirming diffusion-controlled kinetic features; (B) Linear plot of peak current versus square root of scan rate, further substantiating diffusion-controlled behavior; and (C) Chronoamperometric Cottrell plot of peak current versus inverse square root of time ($t^{-1/2}$).

**Fig. S8.** Electrochemical reaction mechanism studies of VON electrooxidation at the CQDs/CPE interface: (A) Plot of anodic peak potential ($E_{p}$) versus pH, demonstrating proton involvement in the electrode process; and (B) Dependence of peak potential ($E_{p}$) on $\log\nu$ according to Laviron’s model, used for the evaluation of electron-transfer kinetics.

**Fig. S9.** DPV voltammograms of VON recorded in $0.04\text{ M}$ BRB across the acidic region (pH 2-6) alongside the optimal condition (pH 9), demonstrating the absence of oxidation signals under acidic conditions compared to the prominent oxidation peak at pH 9.

**Fig. S10.** Influence of the supporting electrolyte on the voltammetric response of VON at the CQDs/CPE surface at pH 9: (A) Overlaid raw DPV voltammograms recorded in $0.04\text{ M}$ BRB and 0.05 M borate buffer; (B) Quantitative comparison of the corresponding anodic peak currents ($I_{p}$).

**Fig. S11.** Voltammetric responses of CQD/CPE: (A) CV and (B) DPV in blank solution and in the presence of VON.

**Fig. S12.** Effect of CQDs loading volume on the anodic peak current response of VON at CQDs/CPE.

**Fig. S13.** Electrochemical response of 50 nM VON: (A) effect of DPV scan rate (5–120 mV s⁻¹) and (B) influence of pulse amplitude (10–100 mV) on the oxidation peak current.

**Fig. S14.** Residual plot for the calibration curve of VON at CQD/CPE showing random distribution around zero.

**Fig. S15.** Representative DPV of VON recorded at the CQDs/CPE interface in Britton–Robinson buffer (0.04 M BR, pH 9) for analytical method validation: (A) Accuracy assessment evaluated at three concentration levels (15, 45, 85 nM), each recorded in triplicate (Trail 1–3); (B) Intraday precision evaluated at 20, 50, and 80 nM, each recorded in triplicate (Trail 1–3); and (C) Interday precision evaluated at 20, 50, and 80 nM across three consecutive days, each recorded in triplicate (Trail 1–3).

**Fig. S16.** CQDs/CPE performance for VON: (A) Short-term stability and (B) corresponding DPVs, (C) long-term stability and (D) corresponding DPVs, (E) reusability and (F) corresponding DPVs, (G) reproducibility and (H) corresponding DPVs.

**Fig. S17.** Selectivity of the CQDs/CPE sensor toward VON: (A) DPV response in a multicomponent mixture, (B–F) individual DPV curves with each co-existing species, and (G) quantitative relative peak current response (n = 3).

**Table S1.** Assessment of method robustness with deliberate parameter changes.

| Parameter | Conditions | %Recovery | Mean %Recovery | | SD | %RSD |
| --- | --- | --- | --- | --- | --- | --- |
| Step Potential | 4 | 99.13 | | 99.31 | 0.4 | 0.4 |
|  | 5 | 99.87 | |  |  |  |
|  | 6 | 98.94 | |  |  |  |
| Pulse Amplitude | 45  50  55 | 100.28  99.64  98.95 | | 99.62 | 0.54 | 0.54 |
| Scan rate | 90 | 101.02 | | 100.48 | 0.66 | 0.66 |
|  | 100  110 | 100.87  99.56 | |  |  |  |
| pH | 8.5 | 99.29 | | 100.04 | 0.62 | 0.62 |
|  | 9 | 100.82 | |  |  |  |
|  | 9.5 | 100.02 | |  |  |  |

**Table S2.** Analytical performance of the CQDs/CPE sensor for VON quantification in commercial dosage forms.

| Conc. (nM) | Response | Found (nM) | %Recovery | Mean %Recovery | SD | %RSD |
| --- | --- | --- | --- | --- | --- | --- |
| 20 | 2.499 | 19.94 | 99.7 | 99.77 | 0.41 | 0.41 |
|  | 2.51 | 20.06 | 100.3 |  |  |  |
|  | 2.492 | 19.86 | 99.3 |  |  |  |
| 40 | 4.49 | 40.33 | 100.83 | 100.05 | 0.56 | 0.56 |
|  | 4.45 | 39.92 | 99.8 |  |  |  |
|  | 4.44 | 39.81 | 99.53 |  |  |  |
| 60 | 6.42 | 60.08 | 100.13 | 99.85 | 0.21 | 0.21 |
|  | 6.4 | 59.88 | 99.8 |  |  |  |
|  | 6.39 | 59.77 | 99.62 |  |  |  |

**Table S3.** Determination of VON in spiked human plasma samples using the CQDs/CPE voltammetric method.

| Conc. (nM) | Response | Found (nM) | %Recovery | Mean %Recovery | SD | %RSD |
| --- | --- | --- | --- | --- | --- | --- |
| 25 | 2.98 | 24.87 | 99.48 | 100.44 | 0.69 | 0.69 |
|  | 3.02 | 25.28 | 101.12 |  |  |  |
|  | 3.01 | 25.18 | 100.72 |  |  |  |
| 55 | 5.97 | 55.48 | 100.87 | 100.37 | 0.47 | 0.47 |
|  | 5.95 | 55.27 | 100.49 |  |  |  |
|  | 5.91 | 54.86 | 99.75 |  |  |  |
| 75 | 7.86 | 74.82 | 99.76 | 99.81 | 0.28 | 0.28 |
|  | 7.84 | 74.62 | 99.49 |  |  |  |
|  | 7.89 | 75.13 | 100.17 |  |  |  |

**Table S4.** Analytical evaluation of the CQDs/CPE sensor for VON quantification in simulated industrial wastewater matrices.

| Conc. (nM) | Response | Found (nM) | %Recovery | Mean %Recovery | SD | %RSD |
| --- | --- | --- | --- | --- | --- | --- |
| 30 | 3.5 | 30.19 | 100.63 | 99.51 | 0.84 | 0.84 |
|  | 3.46 | 29.79 | 99.3 |  |  |  |
|  | 3.44 | 29.58 | 98.6 |  |  |  |
| 50 | 5.45 | 50.15 | 100.3 | 99.97 | 0.25 | 0.25 |
|  | 5.43 | 49.95 | 99.9 |  |  |  |
|  | 5.42 | 49.85 | 99.7 |  |  |  |
| 70 | 7.41 | 70.21 | 100.3 | 99.71 | 0.43 | 0.43 |
|  | 7.36 | 69.7 | 99.57 |  |  |  |
|  | 7.34 | 69.49 | 99.27 |  |  |  |
